# Supplementary material for: Morphological diversity of single neurons in molecularly defined cell types
Source: Nature. 2021 Oct 6;598(7879):174–81. doi: 10.1038/s41586-021-03941-1 (PMC8494643; doi:10.1038/s41586-021-03941-1)

nature portfolio

https://doi.org/10.1038/s41586-021-03941-1

# Supplementary information

# Morphological diversity of single neurons in molecularly defined cell types

In the format provided by the authors and unedited

**Supplementary Figure 1. Overview of reconstructed cortical L5 ET neurons, visualized within the CCFv3 3D reference space.** The first number in the label of each neuron indicates the neuron's cluster assignment shown in Figure 3. Each neuron is shown in two views: top, a coronal plane showing the location of the soma (red dot); bottom, a sagittal maximum projection view showing the brain-wide axon projection pattern. Cortical target regions with axon length >1 mm are indicated by different colors while other axon branches are shown in white.

Supplementary Figure 1 L5 ET cells

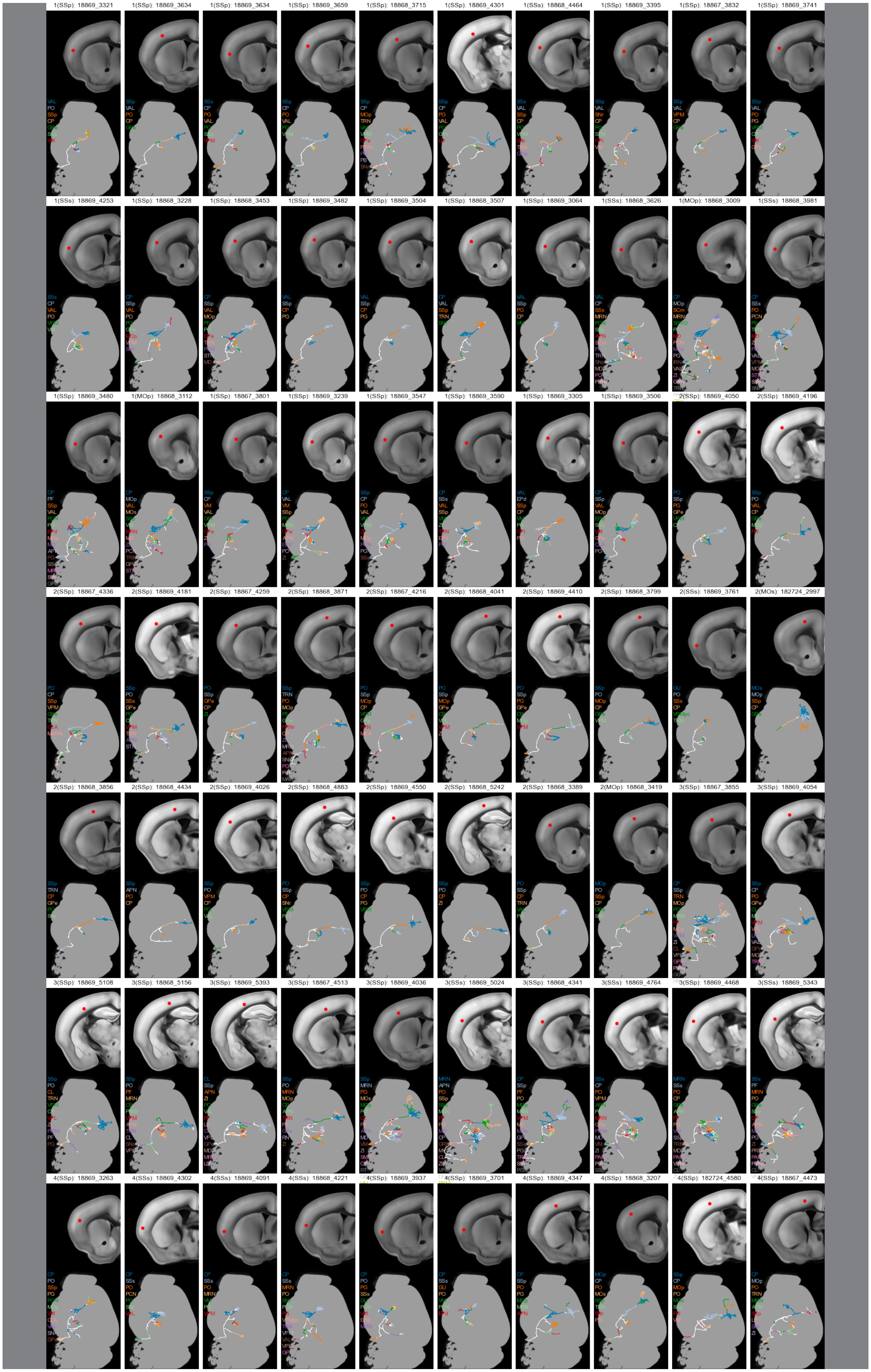

# Supplementary Figure 1 L5 ET cells

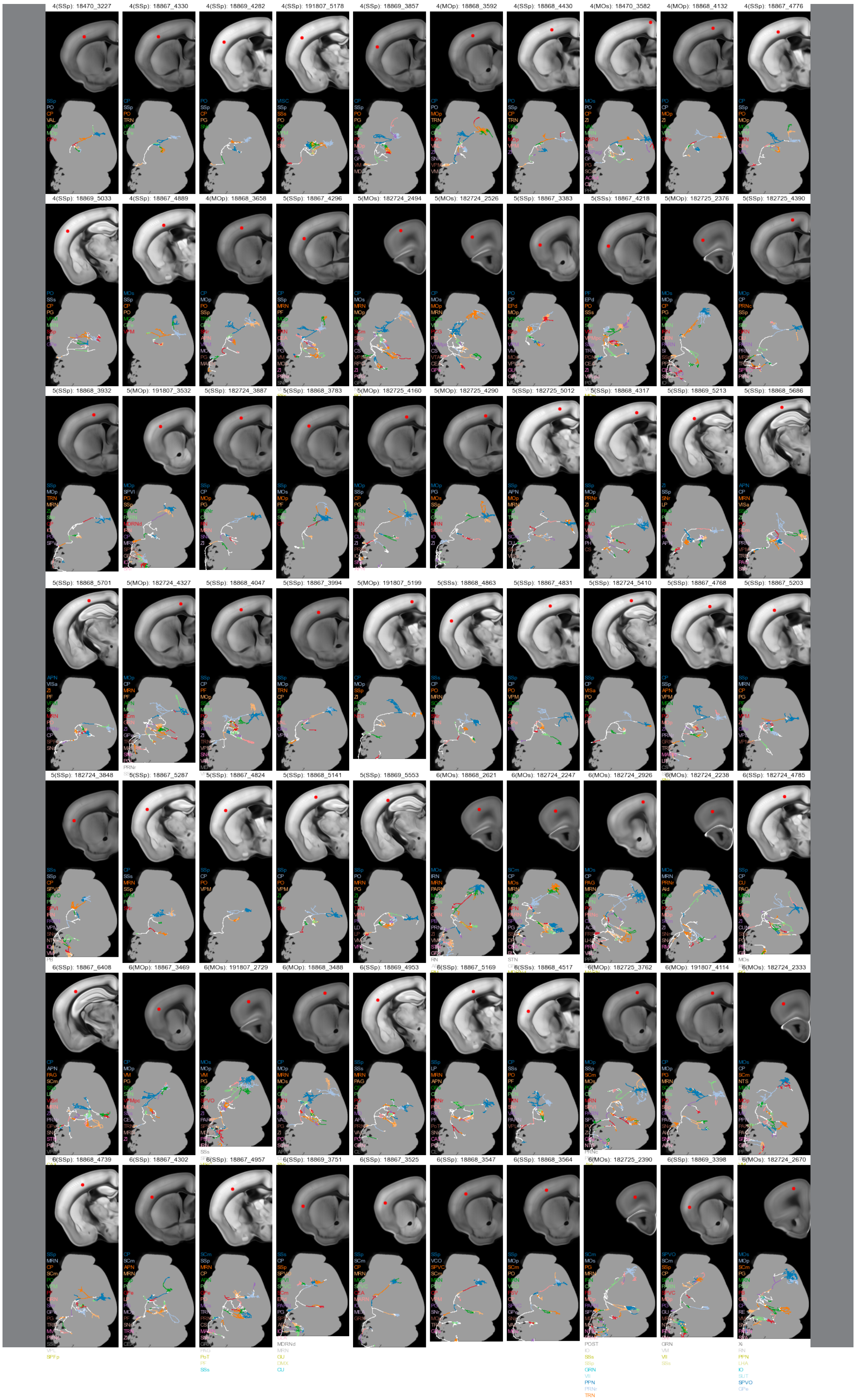

Supplementary Figure 1 L5 ET cells

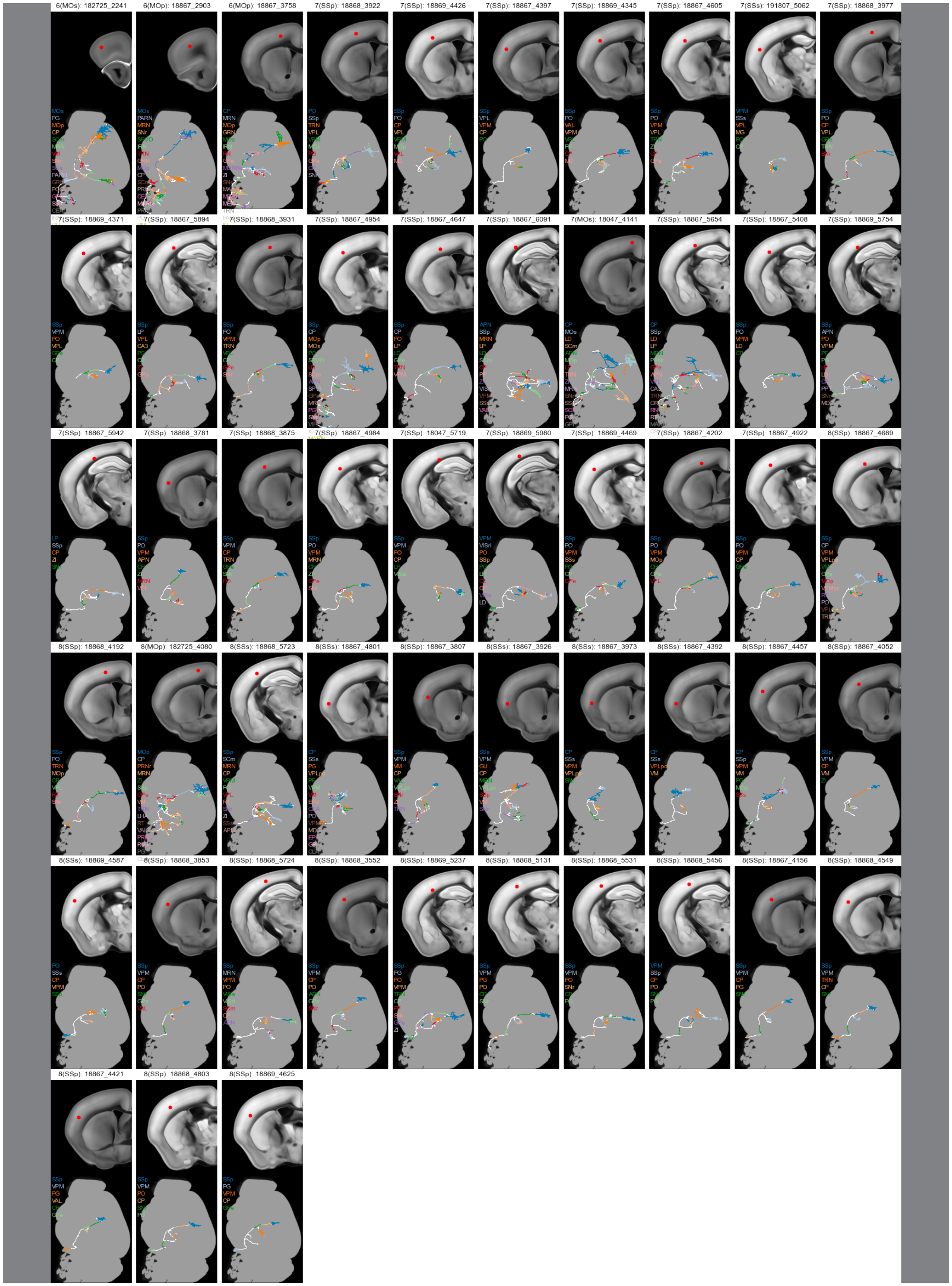

**Supplementary Figure 2. Tri-views of representative reconstructed neurons from core thalamic nuclei, visualized within the CCFv3 3D reference space.** Each tri-view contains three views of the same neuron ordered from top to bottom: a whole-brain top-down view (soma indicated by a star, axon in red), a coronal plane showing the location of the soma (red dot), and a chosen coronal or horizontal plane close to the centre of the main axon arbor with superimposed maximum projection view of the axon arbors. Cortical target regions with axon length >1 mm are indicated by different colours while other axon branches are shown in white.

# Supplementary Figure 2 representative TH core cells

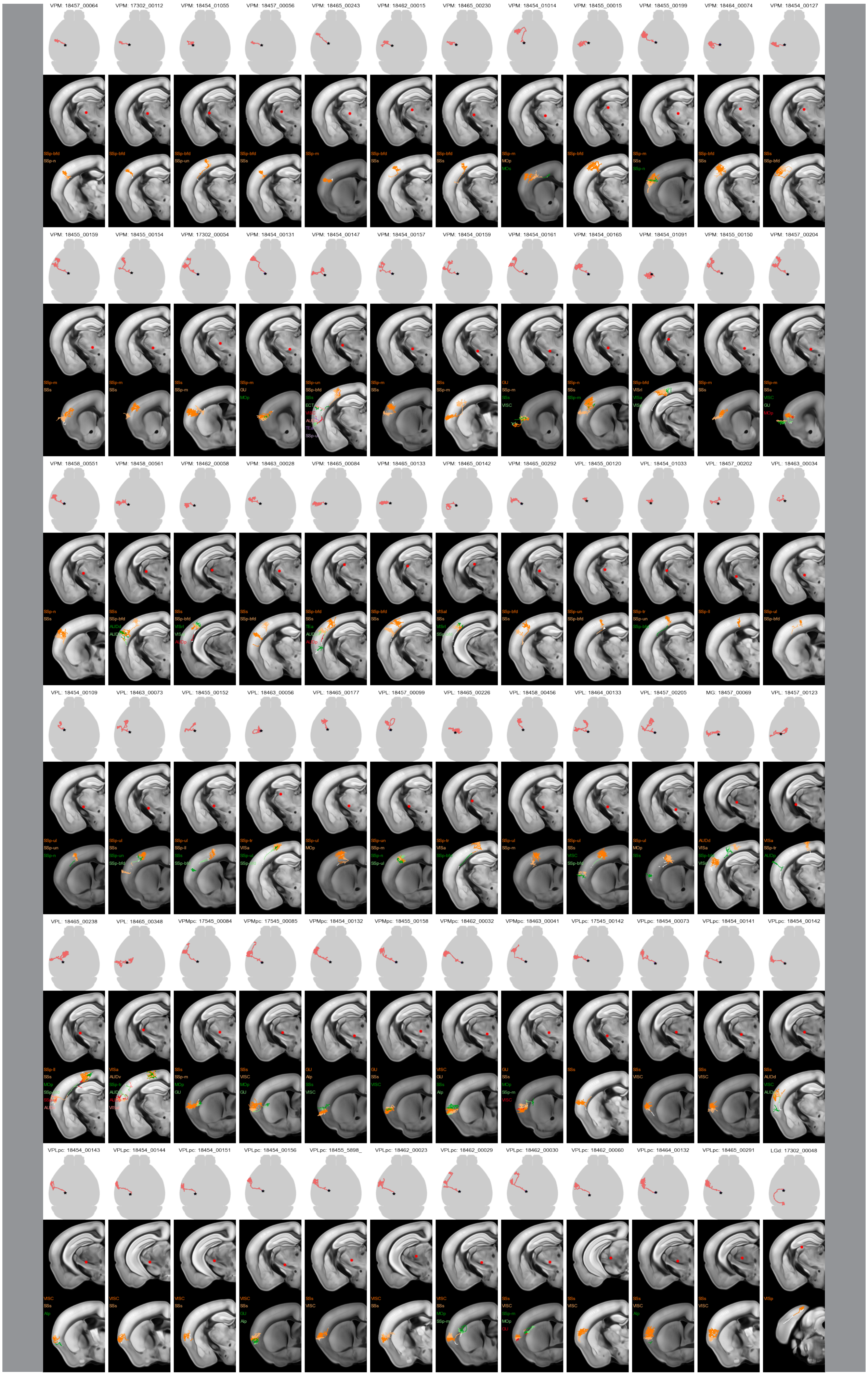

Supplementary Figure 2 representative TH core cells

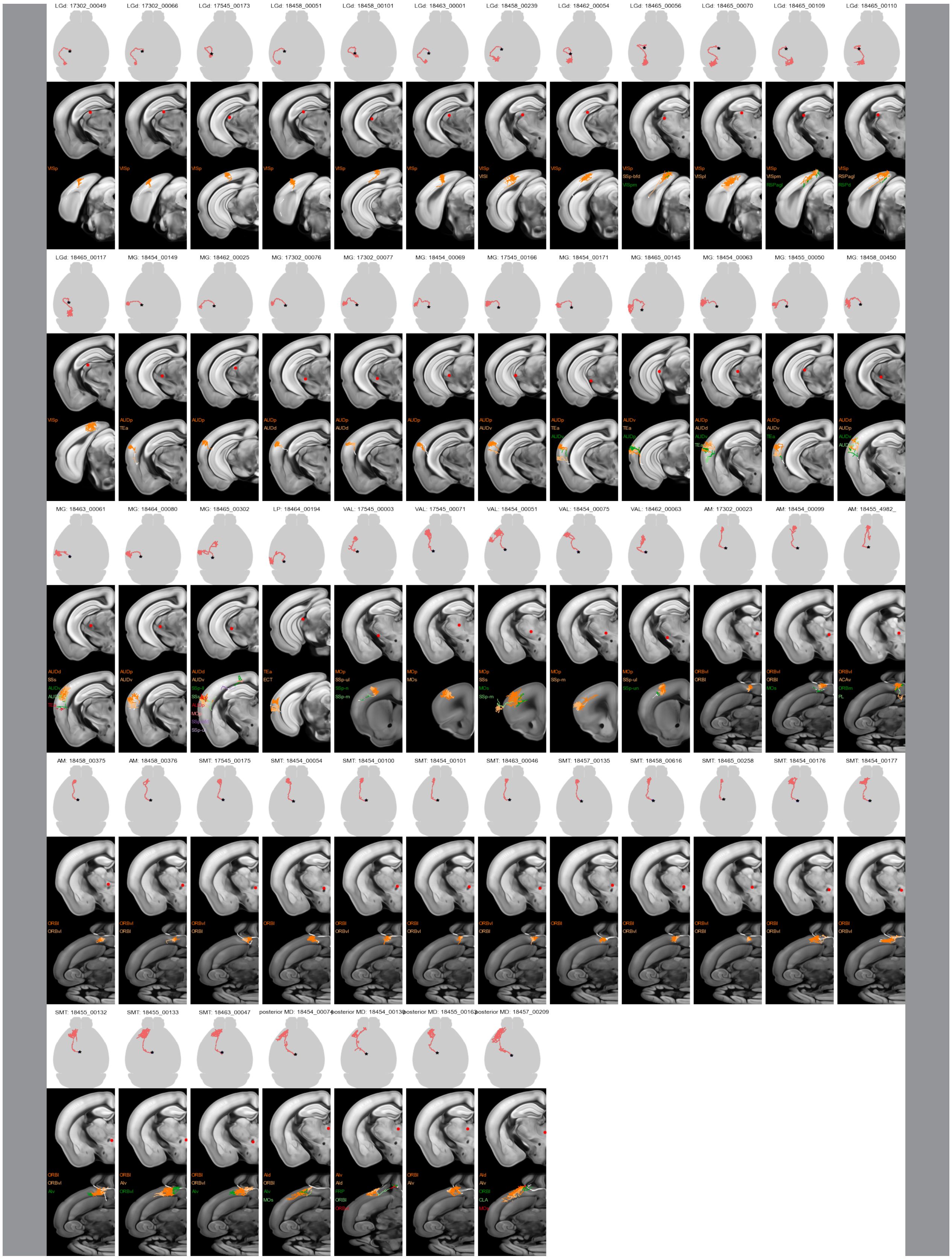

**Supplementary Figure 3. Tri-views of all reconstructed neurons from matrix thalamic nuclei, visualized within the CCFv3 3D reference space.** Each tri-view contains three views of the same neuron ordered from top to bottom: a whole-brain top-down view (soma indicated by a star, axon in red), a coronal plane showing the location of the soma (red dot), and a chosen coronal plane close to the centre of the main axon arbor with superimposed maximum projection view of the axon arbors. Cortical target regions with axon length >1 mm are indicated by different colours while other axon branches are shown in white.

# Supplementary Figure 3 all TH matrix cells

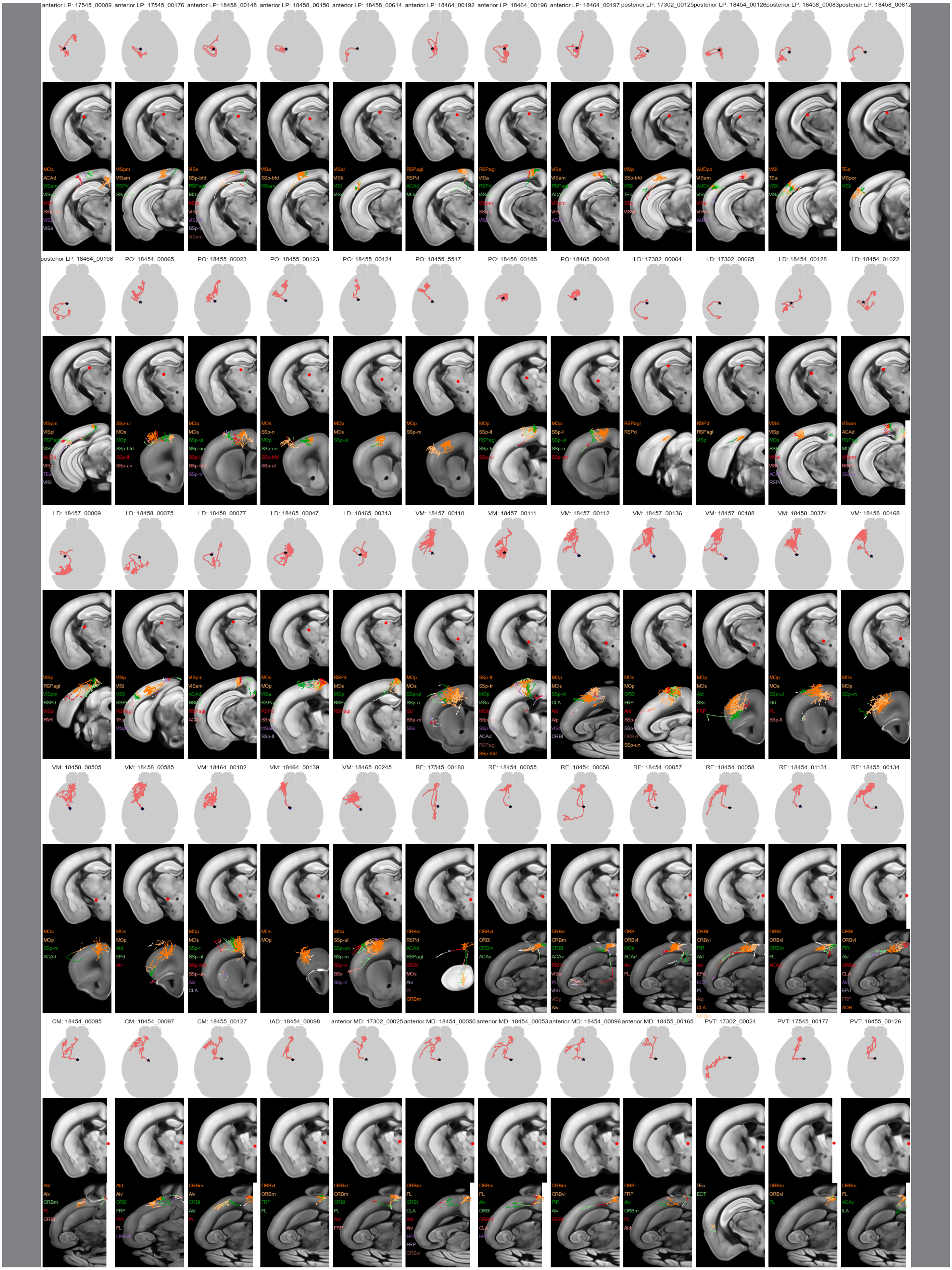

Supplement: Supplementary file 1 — Supplementary Figs. 1–3. [file 41586_2021_3941_MOESM1_ESM.pdf]
