# Supplementary material for: Morphological diversity of single neurons in molecularly defined cell types
Source: Nature. 2021 Oct 6;598(7879):174–81. doi: 10.1038/s41586-021-03941-1 (PMC8494643; doi:10.1038/s41586-021-03941-1)
Supplement: Supplementary file 2 — Reporting Summary [file 41586_2021_3941_MOESM2_ESM.pdf]

nature research

Corresponding author(s): Hanchuan Peng, Hongkui Zeng

Last updated by author(s): Aug 15, 2021

Reporting Summary

Nature Research wishes to improve the reproducibility of the work that we publish. This form provides structure for consistency and transparency in reporting. For further information on Nature Research policies, see our Editorial Policies and the Editorial Policy Checklist.

Statistics

For all statistical analyses, confirm that the following items are present in the figure legend, table legend, main text, or Methods section.

n/a	Confirmed	
☐	☒	The exact sample size (*n*) for each experimental group/condition, given as a discrete number and unit of measurement
☐	☒	A statement on whether measurements were taken from distinct samples or whether the same sample was measured repeatedly
☐	☒	The statistical test(s) used AND whether they are one- or two-sided<br>*Only common tests should be described solely by name; describe more complex techniques in the Methods section.*
☐	☒	A description of all covariates tested
☒	☐	A description of any assumptions or corrections, such as tests of normality and adjustment for multiple comparisons
☐	☒	A full description of the statistical parameters including central tendency (e.g. means) or other basic estimates (e.g. regression coefficient) AND variation (e.g. standard deviation) or associated estimates of uncertainty (e.g. confidence intervals)
☐	☒	For null hypothesis testing, the test statistic (e.g. *F*, *t*, *r*) with confidence intervals, effect sizes, degrees of freedom and *P* value noted<br>*Give P values as exact values whenever suitable.*
☒	☐	For Bayesian analysis, information on the choice of priors and Markov chain Monte Carlo settings
☒	☐	For hierarchical and complex designs, identification of the appropriate level for tests and full reporting of outcomes
☒	☐	Estimates of effect sizes (e.g. Cohen's *d*, Pearson's *r*), indicating how they were calculated

Our web collection on statistics for biologists contains articles on many of the points above.

Software and code

Policy information about availability of computer code

Data collection	TissueCyte 1000, fMOST and Vaa3D were used in data acquisition processes
Data analysis	Vaa3D (version 3.604), TeraFly (version 2.5.101) and TeraVR (version bundled with Vaa3D) are available at Vaa3D's github site, https://github.com/Vaa3D, with both source code and binary executable. The mBrainAligner package is available via https://github.com/Vaa3D/vaa3d_tools/blob/master/hackathon/mBrainAligner.<br>Python package neuro_morpho_toolbox (https://github.com/pengxie-bioinfo/neuro_morpho_toolbox) is used for full morphology analysis. Custom data analysis notebooks are available via https://github.com/pengxie-bioinfo/BICCN_full_morphology.<br>STAR v2.5.3 and R GenomicAlignments package (RRID: SCR_018096) are used for RNA-seq alignment. R package scrattch.hicat (https://github.com/AllenInstitute/scrattch.hicat) is used for scRNA-seq analysis, including mapping retro-seq cells to reference taxonomy and re-clustering.

For manuscripts utilizing custom algorithms or software that are central to the research but not yet described in published literature, software must be made available to editors and reviewers. We strongly encourage code deposition in a community repository (e.g. GitHub). See the Nature Research guidelines for submitting code & software for further information.

Data

Policy information about availability of data

All manuscripts must include a data availability statement. This statement should provide the following information, where applicable:

- Accession codes, unique identifiers, or web links for publicly available datasets
- A list of figures that have associated raw data
- A description of any restrictions on data availability

Data availability statement is provided in the manuscript.

The fMOST image datasets (https://download.brainimagelibrary.org/biccn/zeng/luo/fMOST/) of all mouse brains used in this study, as well as the original and CCFv3 registered single neuron reconstructions (DOI: https://doi.org/10.35077/g.25), are available at BICCN's Brain Image Library (BIL) at Pittsburgh Supercomputing Center (www.brainimagelibrary.org). The single neuron reconstructions, the CCFv3 registered version of these reconstructions, as well as 3D navigation movie-gallery of these data are available at SEU-ALLEN Joint Center, Institute for Brain and Intelligence (https://braintell.org/projects/fullmorpho/).
Mesoscale AAV-tracing data (including high resolution images, segmentation, registration to CCFv3, and automated quantification of injection size, location, and distribution across brain structures) are available through the Allen Mouse Brain Connectivity Atlas portal (http://connectivity.brain-map.org/). Expression patterns of transgenic mouse lines can be found in the Allen Transgenic Characterization database (http://connectivity.brain-map.org/transgenic/search/basic). Retro-seq SMART-Seq v4 data are available at the NCBI Gene Expression Omnibus (GEO) under accession GSE181363.

Field-specific reporting

Please select the one below that is the best fit for your research. If you are not sure, read the appropriate sections before making your selection.

☒ Life sciences ☐ Behavioural & social sciences ☐ Ecological, evolutionary & environmental sciences

For a reference copy of the document with all sections, see nature.com/documents/nr-reporting-summary-flat.pdf

Life sciences study design

All studies must disclose on these points even when the disclosure is negative.

Sample size	No sample size calculation was performed. Sample sizes were determined by cell type specificity of mouse lines and limited by practical reasons, such as imaging quality and sparsity of labeling. Sample sizes are provided in Supplementary Tables 1 and 3. Specifically, 1-7 brains each transgenic mouse line used for generating neuron reconstructions for analysis in the current study had 3-7 whole-brain fMOST imaging datasets. Dozens to hundreds of cells were reconstructed from each selected brain.
Data exclusions	Full morphology dataset generated by this study has been made available. Low quality cells (weak labeling or too crowded labeling) that can't be fully reconstructed were excluded from reconstruction. For comparative analysis with mesoscale data, we excluded mesoscale experiments with injection contamination in neighboring brain regions.<br>All Retro-seq cells in the targeted regions were included for analysis.
Replication	Each mouse line used for analysis in the current study contained at least three mice for imaging data acquisition. For analysis of projection patterns, a minimum of three single cells was required for each cell type in each brain region. Each neuron reconstruction was validated by at least 2 or 3 annotators in the workflow. All attempts at replication were successful.
Randomization	This study did not involve allocation of experimental groups. Data were grouped by Cre-lines or soma locations. Samples were not randomized.
Blinding	Blinding is not applicable to the study design. There was no allocation of treatment and control groups.

Reporting for specific materials, systems and methods

We require information from authors about some types of materials, experimental systems and methods used in many studies. Here, indicate whether each material, system or method listed is relevant to your study. If you are not sure if a list item applies to your research, read the appropriate section before selecting a response.

Materials & experimental systems

n/a	Involved in the study	
☒	☐	Antibodies
☒	☐	Eukaryotic cell lines
☒	☐	Palaeontology and archaeology
☐	☒	Animals and other organisms
☒	☐	Human research participants
☒	☐	Clinical data
☒	☐	Dual use research of concern

Methods

n/a	Involved in the study	
☒	☐	ChIP-seq
☒	☐	Flow cytometry
☒	☐	MRI-based neuroimaging

Animals and other organisms

Policy information about studies involving animals; ARRIVE guidelines recommended for reporting animal research

Laboratory animals	We used transgenic mice that contain a combination of the following individual driver and reporter lines: Cux2-CreERT2, Fezf2-CreER, Gnb4-IRES2-CreERT2, Plxnd1-CreER, Pvalb-T2A-CreERT2, Tnnt1-IRES2-CreERT2, Vipr2-IRES2-Cre-neo, Snap25-IRES2-Cre, Slc17a7-IRES2-Cre, Esr2-IRES2-Cre, Ai139, Ai140, Ai82, Ai166, Ai14, Ai65F, and RCL-Sun1sfGFP. All transgenic mice were maintained in C57BL/6J congenic background. For each genotype of transgenic mice, we used both male and female mice, ages ranging from 8 weeks to 5 months old.

	Mice were housed in animal rooms on a 14/10 hr light/dark cycle (6am-8pm light). The room temperature was set at 70°F (21°C) and the relative humidity at 40%.
Wild animals	The study did not involve wild animals.
Field-collected samples	The study did not involve samples collected from the field.
Ethics oversight	All experimental procedures using live animals were performed according to protocols approved by Institutional Animal Care and Use Committee (IACUC) of the Allen Institute for Brain Science.

Note that full information on the approval of the study protocol must also be provided in the manuscript.
